# Supplementary material for: Enantioseparation of Proton Pump Inhibitors by HPLC on Polysaccharide-Type Stationary Phases: Enantiomer Elution Order Reversal, Thermodynamic Characterization, and Hysteretic Effect
Source: Int J Mol Sci. 2025 Jul 25;26(15):7217. doi: 10.3390/ijms26157217 (PMC12346594; doi:10.3390/ijms26157217)
Supplement: Supplementary file 1 [file ijms-26-07217-s001.zip › ijms-3716787-supplementary.pdf]

SUPPLEMENTARY MATERIALS

TO

Enantioseparation of proton pump inhibitors by HPLC on polysaccharide-type stationary phases: enantiomer elution order reversal, thermodynamic characterization and hysteretic effect

Máté Dobó [1,2], Gergely Molnár [1,2], Ali Mhammad [1,2], Gergely Dombi [1,2], Zoltán-István Szabó [3,4] and Gergő Tóth [1,2,*]

[1] Department of Pharmaceutical Chemistry, Semmelweis University, Hőgyes E. str. 9, H-1092 Budapest, Hungary;

[2] Center for Pharmacology and Drug Research & Development, Semmelweis University, Budapest, Hungary

[3] Department of Pharmaceutical Industry and Management, George Emil Palade University of Medicine, Pharmacy, Science and Technology of Targu Mures, Gh. Marinescu 38, 540139 Targu Mures, Romania;

[4] Sz-imfidum Ltd., Lunga nr. 504, 525401 Lunga, Romania

*Correspondence: toth.gergo@semmelweis.hu

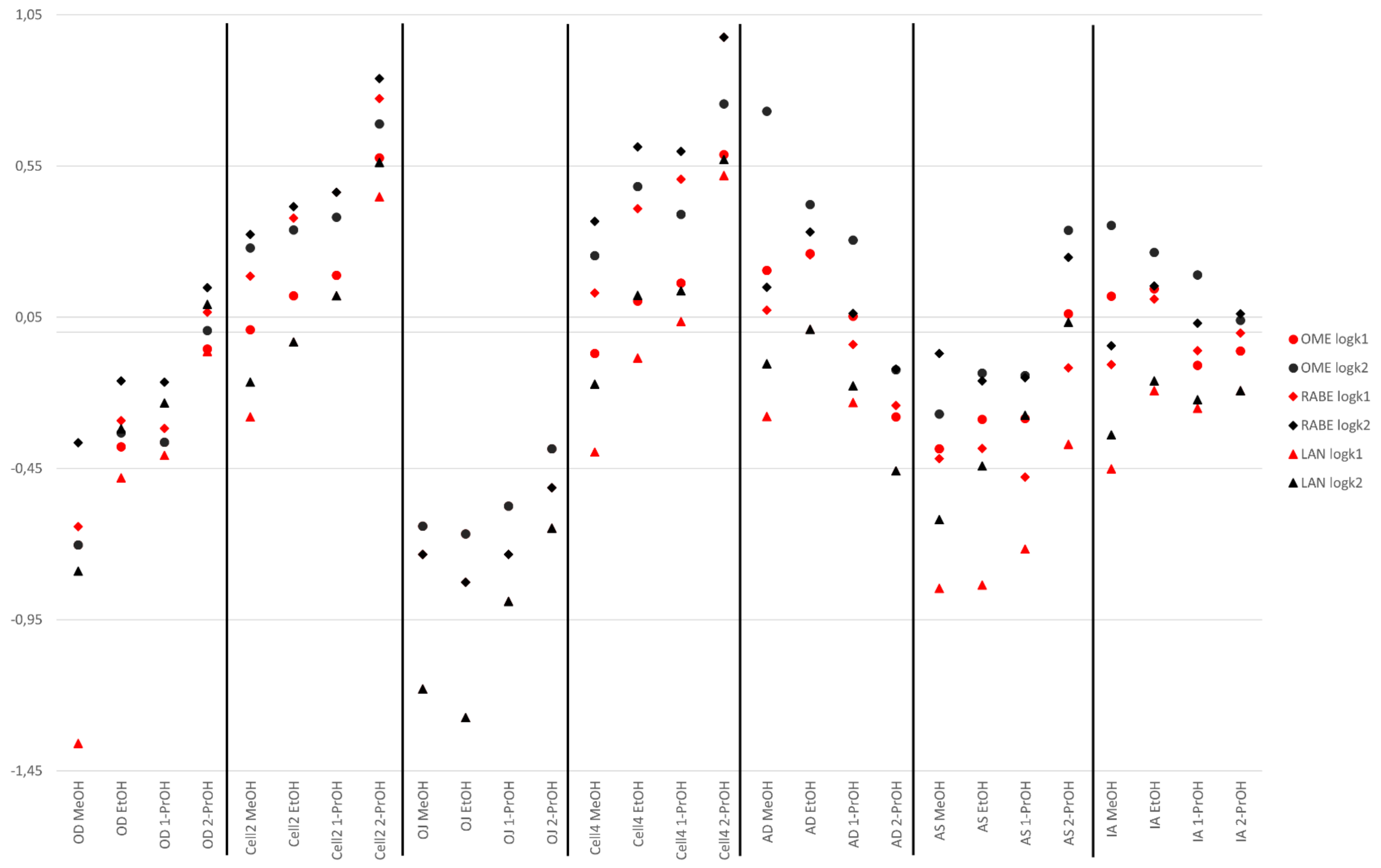

Supplementary Figure 1. logk values in different selector-mobile phase systems (OME-omeprazole, RABE-rabeprazole, LAN-lansoprazole)

Supplementary Table 1. Calculated Thermodynamic data

CSP	Mobile phase	Omeprazole					Rabeprazole					Lansoprazole				
		Δ(ΔH°) (kJ/mol)	Δ(ΔS°) (J/molK)	Δ(ΔG°) (kJ/mol)	T_{iso} (°C)	*Q*	Δ(ΔH°) (kJ/mol)	Δ(ΔS°) (J/molK)	Δ(ΔG°) (kJ/mol)	T_{iso} (°C)	*Q*	Δ(ΔH°) (kJ/mol)	Δ(ΔS°) (J/molK)	Δ(ΔG°) (kJ/mol)	T_{iso} (°C)	*Q*
Chiralpak AD	MeOH	5.95	30.24	-15.50	-489.00	0.66	1.54	6.65	-0.43	-40.7	0.78	1.44	8.17	-0.99	-95.83	0.59
	EtOH	1.7	8.68	-0.43	-77.00	0.66	-5.43	-17.32	-0.28	40.92	1.05	-	-	-	-	-
	1-PrOH	2.45	12.99	-1.42	-84.44	0.63	4.21	16.12	-0.60	-11.93	0.88	2.79	10.41	-0.31	-4.92	0.90
	2-PrOH	-7.31	-2232.00	-0.66	54.44	1.10	-2.20	-5.69	-0.50	113.74	1.30	-	-	-	-	-
Chiralpak IA	MeOH	7.04	28.81	-1.55	-28.84	0.82	0.44	2.73	-0.37	-110.13	0.55	-0.81	-0.51	-0.67	1336.56	5.40
	EtOH	1.699	7.89	-0.65	-57.82	0.72	-4.65	-15.055	-0.17	36.01	1.04	-4.65	-14.91	-0.17	38.90	1.05
	1-PrOH	5.57	23.06	-1.31	-31.59	0.81	4.13	15.19	-0.40	-1.07	0.91	-	-	-	-	-
	2-PrOH	-0.74	-0.59	-0.57	992.89	4.25	0.623	3.39	-0.39	-89.09	0.62	-	-	-	-	-
Chiralpak AS	MeOH	-5.88	-17.91	-0.55	55.73	1.10	-7.44	-18.9	-1.81	120.99	1.32	-5.91	-15.91	-1.17	98.59	1.25
	EtOH	1.37	7.77	-0.95	-96.58	0.59	5.05	22.07	-1.52	-43.95	0.77	4.76	23.51	-2.25	-70.57	0.68
	1-PrOH	2.13	9.66	-0.75	-52.45	0.74	2.142	13.31	-1.82	-112.01	0.54	3.06	18.96	-2.59	-111.66	0.54
	2-PrOH	-2.85	-4.16	-1.62	413.41	2.30	-2.26	-0.22	-2.20	9833.82	33.92	-2.52	-0.33	-2.43	7465.53	25.97
Chiralcel OD	MeOH	-	-	-	-	-	-2.98	-7.56	-0.73	121.83	1.32	-2.91	-7.15	-0.79	135.25	1.37
	EtOH	-1.68	-5.36	-0.08	40.20	1.05	-1.65	-3.73	-0.54	169.92	1.49	-1.97	-4.36	-0.67	178.65	1.52
	1-PrOH	-	-	-	-	-	-2.16	-4.87	-0.71	171.40	1.49	-2.11	-4.44	-0.79	201.84	1.59
	2-PrOH						-	-	-	-	-	-2.24	-5.93	-0.47	104.75	1.27
Lux Cell2	MeOH	-1.75	-0.69	-1.52	2225.36	8.38	-0.88	-0.69	-0.77	2056.90	7.82	1.15	6.05	-0.65	-82.81	0.64
	EtOH	-1.714	-1.70	-1.21	734.43	3.38	2.53	9.05	-0.17	6.35	0.94	-	-	-	-	-
	1-PrOH	-1.17	-0.30	-1.09	3676.79	13.25	-	-	-	-	-	-	-	-	-	-
	2-PrOH	11.86	41.99	-0.65	9.41	0.95	-	-	-	-	-	-	-	-	-	-
Lux Cell4	MeOH	-1.62	-0.16	-1.58	9793.80	33.78	-3.53	-7.49	-1.29	196.90	1.58	-4.34	-10.37	-1.25	145.86	1.41
	EtOH	-1.95	-1.88	-1.39	762.24	3.47	1.16	6.06	-0.65	-82.15	0.64	2.67	10.71	-0.52	-23.63	0.84
	1-PrOH	0.34	2.75	-0.48	-149.00	0.42	0.82	4.33	-0.47	-82.92	0.64	1.75	7.25	-0.42	-32.34	0.81
	2-PrOH	1.37	7.01	-0.71	-76.31	0.66	0.38	1.34	-0.01	10.50	0.95	-	-	-	-	-

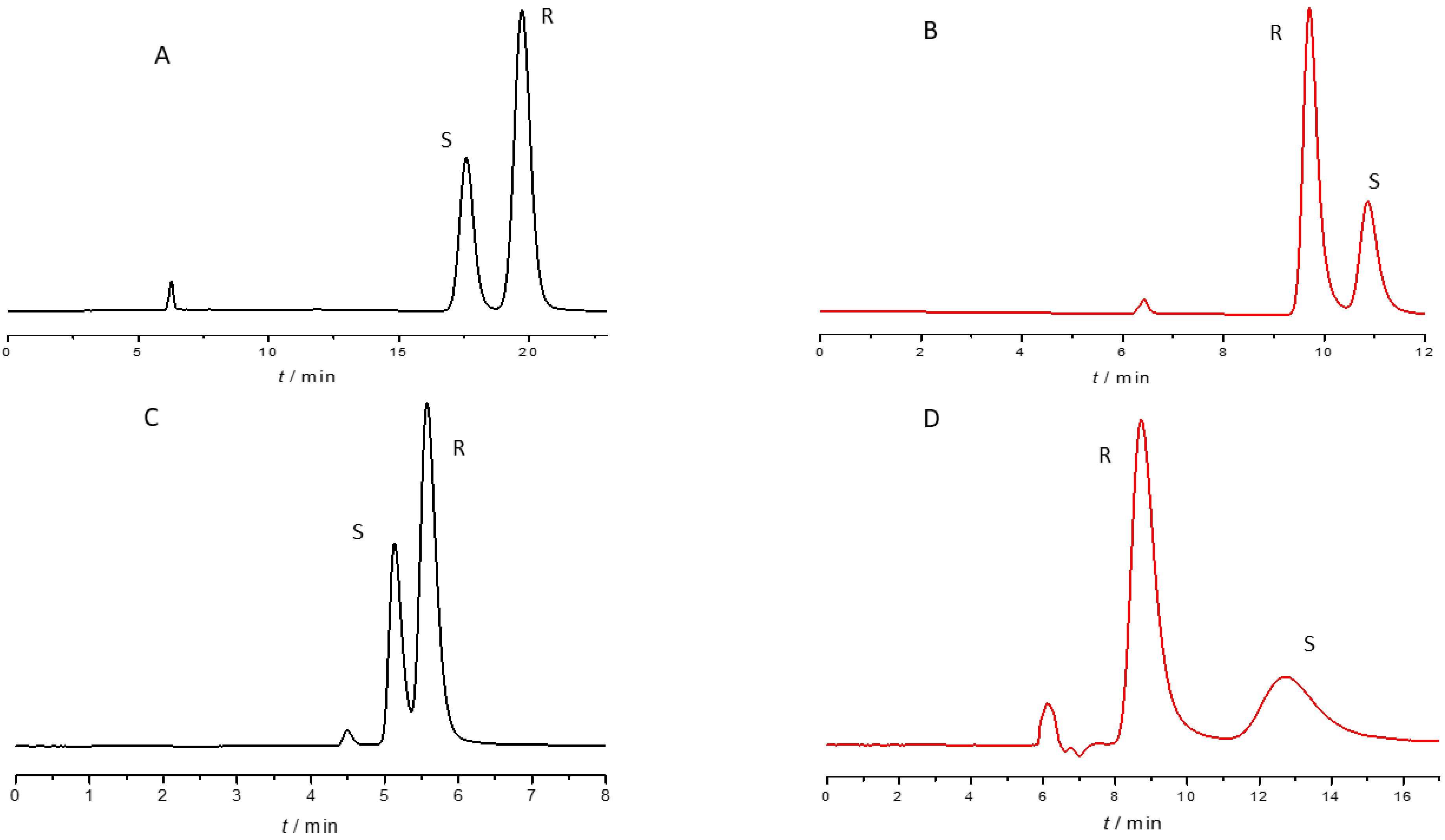

Supplementary Figure 2 Representative chromatograms illustrating the enantiomeric elution order. A – Rabeprazol Chiralpak AD column with ethanol, B – Rabeprazol Chiralcel OD column with ethanol, C – lansoprazole Chiralpak AS column with methanol, D – Chiralpak AS column with 2-propanol
